# Reproducibility of a protocol for standardized reading of chest X-rays of children household contact of patients with tuberculosis

**DOI:** 10.1186/s12887-022-03347-6

**Published:** 2022-05-24

**Authors:** María Margarita Lozano-Acosta, María Alejandra Rubiano-Arenas, Lina Marcela Cadavid, Guillermo Vélez-Parra, Beatriz Molinares, Diana Marcela Marín-Pineda, María Patricia Arbeláez-Montoya, Dione Benjumea-Bedoya

**Affiliations:** 1 Grupo de Investigación en Salud Familiar y Comunitaria, Facultad de Ciencias de la Salud, Corporación Universitaria Remington, Calle 51 # 51-27, Medellín, Colombia; 2 Pablo Tobón Uribe Hospital - IMEDI, Medellín, Colombia; 3 Hospital General de Medellín, Docente Universidad CES, Medellín, Colombia; 4 Hospital Pablo Tobón Uribe, Medellín, Colombia; 5 Grupo de Investigación en Salud Pública, Universidad Pontificia Bolivariana, Medellín, Colombia; 6 Grupo de Epidemiología, Facultad Nacional de Salud Pública, Universidad de Antioquia, Medellín, Colombia; 7 Grupo de Bacteriología y Micobacterias, Corporación Para Investigaciones Biológicas-CIB, Medellín, Colombia

**Keywords:** Tuberculosis, Pulmonary, Latent Tuberculosis, Clinical Protocols, Radiography, Thoracic, Observer Variation, Reproducibility of Results

## Abstract

**Background:**

The interpretation of the chest radiograph may vary because it depends on the reader and due to the non-specificity of findings in tuberculosis (TB). We aim to assess the reproducibility of a standardized chest radiograph reading protocol in contacts of patients with pulmonary TB under the 5 years of age.

**Methods:**

Descriptive, cross-sectional study with children under the age of five, household contacts of patients with confirmed pulmonary TB from Medellín, Bello and Itagüí (Colombia) between Jan-01–2015 and May-31–2016. Standardized reading protocol: two radiologists, blinded independent reading, use of template (Dr. Andronikou design) in case of disagreement a third reading was performed. Kappa coefficient for intra and inter observer agreement, and prevalence ratio were estimated of sociodemographic characteristics, TB exposure and interpretation of chest X-ray.

**Results:**

From 278 children, standardized reading found 255 (91.7%) normal X-rays, 10 (3.6%) consistent with TB, and 13 (4.7%) other alterations. Global agreement was 91.3% (Kappa = 0.51). Inter-observer agreement between readers 1–2 was 90.0% (Kappa = 0.59) and 1–3 93.2% (Kappa = 0.59). Intra-observer agreement for reader 1 was 95.5% (Kappa = 0.86), 2 84.0% (Kappa = 0.51), and 3 94.7% (Kappa = 0.68). Greater inter-observer disagreement was between readers 1–2 for soft tissue density suggestive of adenopathy (4.6%), airspace opacification (1.17%) and pleural effusion (0.58%); between readers 1–3 for soft tissue density suggestive of adenopathy (4.2%), opacification of airspace (2.5%) and cavities (0.8%).

**Conclusions:**

Chest radiographs are an affordable tool that contributes to the diagnosis of TB, so having a standardized reading protocol showed good agreement and improves the reproducibility of radiograph interpretation.

## Background

Tuberculosis (TB) is an infectious disease caused by the *Mycobacterium tuberculosis* complex, which is present throughout the world. It is the ninth leading cause of death, with a predominance in developing countries; where human immunodeficiency virus (HIV) infection is high and resources are insufficient for disease identification and treatment [1–5].

Approximately 10 million people became ill with TB in 2019, 12% were children under the age of 15. Geographically, the majority were in regions of Southeast Asia (44%), East Africa (8.2%), America (2.9%) and Europe (2.5%); furthermore, only 54 countries had a low incidence of TB (less than 10 cases per 100,000 inhabitants per year). Of the 1.3 million household contacts with TB who should access preventive treatment in 2019, only 538,396 (33%) received it, and 81% of them were children under the age of 5 [6].

The strategy to reduce the incidence of active TB in childhood is only efficient when adequate and timely treatment is given for latent TB [6, 7]. Additionally, the diagnosis of TB in pediatrics is difficult, since the symptoms and radiographic changes are less specific at this age [1, 8, 9]. The grouping of factors such as: paucibacillary characteristic of the infection, the laborious collection of respiratory samples, the variable symptoms and the severity of the disease exacerbated by other childhood infections such as pneumonia, malnutrition and HIV; limit the diagnostic approach, resulting in unreliable statistics due to insufficient recognition of the disease [1, 6, 10–12].

The notion of TB diagnosis in children under the age of 5 household contacts with TB patients, is based on the risk of developing active TB, for having contact with the infection at home, for which is necessary to intervene on time to prevent progression to disease [13, 14]. Laboratory tests showing infection are required initially, in addition to a chest X-ray [12]. Having a positive immunological test in the absence of clinical manifestations and a normal chest X-ray, guides the diagnosis of latent TB; which requires treatment to prevent the disease [1, 7, 9, 10, 14].

The radiological signs in the radiographs of patients with TB are not pathognomonic of the disease and may be present in other pulmonary pathologies; leading to a problem in medical practice as it can draw an inappropriate approach to the disease [12, 14–16]. Reading the radiograph is operator dependent, so the experience of the reader is important; since there is the possibility of errors when interpreting it due to the subjectivity that it entails [7, 17, 18]. However, there are characteristic patterns and signs that help the radiologist to give a reliable reading [8]. Therefore, the main objective of this study was to evaluate the reproducibility of a standardized reading protocol for chest radiographs in children with contact of TB, in order to contribute to the accurate diagnosis of TB in pediatrics, reducing the subjectivity of reading, improper diagnosis and ineffective treatments [3, 8, 9, 14, 19, 20].

## Methods

### Design and population

Descriptive, cross-sectional study, with primary and secondary source of chest X-rays from children under the age of five living with patients with bacteriologically confirmed pulmonary TB from Medellín, Bello and Itagüí (Colombia), notified to the epidemiological surveillance system (SIVIGILA) between Jan-01–2015 and May-31–2016.

### Procedures

X-rays were taken at a tertiary hospital and images were read in electronic format. Interpretation of chest x-rays was performed using a standardized protocol which consists of reading the anteroposterior (AP) and lateral chest X-ray by two independent and blinded readers, by using a template, and in case of disagreement, a third reading was done by a different person. In the case of the current study, the readers were a radiologist with a pediatric specialty with 13 years of experience, a general radiologist with an emphasis on thorax with 21 years of experience, and a radiologist with a specialty in body radiology with 18 years of experience. The template used in the study was created by Dr. Savvas Andronikou for educational purposes in South Africa [21], and it consists of the following items to assess during reading: airway compression and/or trachea displacement, soft tissue density suggestive of lymphadenopathy, air space opacification, disseminated and bilateral nodular = miliary or greater image, pleural effusion, cavities, calcified parenchyma (Ghon focus), and vertebral spondylitis. The classification for reading according to the template was: normal (without abnormalities suggestive of current or previous TB), consistent with TB (if there is a positive response to some of the radiological characteristics in the same location by the two experts) and other alterations (findings different from the items described in the template). The quality of chest X-rays (AP and lateral each one) was rated according to three categories: acceptable; poor but readable; not acceptable, not readable. Authorization was obtained for the use of the template in the current study.

### Variables

Following variables were collected throughout a standardized questionnaire: sex, age of the household contact children, city of residence, schooling of the person in charge, socioeconomic status, overcrowding, displacement status, health care affiliation, ethnicity, evidence of Bacille Calmette-Guerin (BCG) vaccine, persistent cough, loss of weight, failure to thrive, fever, lethargy, lymphadenopathy, sweating, diagnosis compatible with active TB, Quantiferon TB-Gold interpretation (QTF), interpretation of the tuberculin skin test (TST), chest X-ray interpretation, initial infection status, and prevalent active TB. To association analysis variables were categorized as follows: age of the household contact children < 12 months or ≥ 12 months; socioeconomic stratum with low stratum (strata 1 and 2) and not low (strata 3, 4 and 5); health care system affiliation in the groups do not have-no data, linked-subsidized and contributory; ethnicity in indigenous and others; proximity to the index case in does not live in the same house and sleeps in the same bed, room or house; evidence of BCG in vaccinated and unvaccinated, no scar - no immunization card, no information.

### Sample size

For inter-observer agreement were included all radiographs performed to children in the study and read by two radiologists (during recruitment period), totaling 278 radiographs. Since reader 1 had previously read all radiographs, it was possible to compare reader 1 to 2, and reader 1 to 3. For the intra-observer agreement analysis, it was considered to read for second time (during 2020) under blindness all X-rays that had disagreement or abnormal interpretation, and to take an aleatory sample of the X-rays that had normal interpretation during the first reading. The sample size was estimated for a difference in the proportions of paired samples (McNemar’s test), with 95% confidence, 80% power, global inter-observer agreement of 93.6%, and a proportion of normal readings between 85 and 90%, resulting in 80 chest X-ray for second reading for each observer.

### Statistical analysis

Sociodemographic and exposure characteristics of the participating children were described throughout frequencies and proportions, and it was analyzed their association with the interpretation of the chest X-ray with the likelihood ratio test. Prevalence ratios (PR) were estimated of having an X-ray consistent with TB with respect to a normal X-ray with their respective 95% confidence interval. With kappa coefficient it was evaluated the global inter-observer agreement, and according to the group’s reader 1—reader 2, and reader 1—reader 3; and it was calculated the proportion of intra-observer agreement for each of the items in the reading template. The kappa coefficient was considered poor (< 0.2), weak (0.21—0.40), moderate (0.41—0.60), good (0.61–0.80), very good (0.81–1) [22]. The analyzes were performed in SPSS 22 and EPIDAT 3.1.

### Ethical aspects

This study was classified without risk and was approved by the Bioethics Committee of the Corporación Universitaria Remington (record September 23, 2019). Written informed consent was obtained from a parent or guardian for all participants. Approved use of the template by Dr. Savvas Andronikou.

## Results

**Fig. 1:**
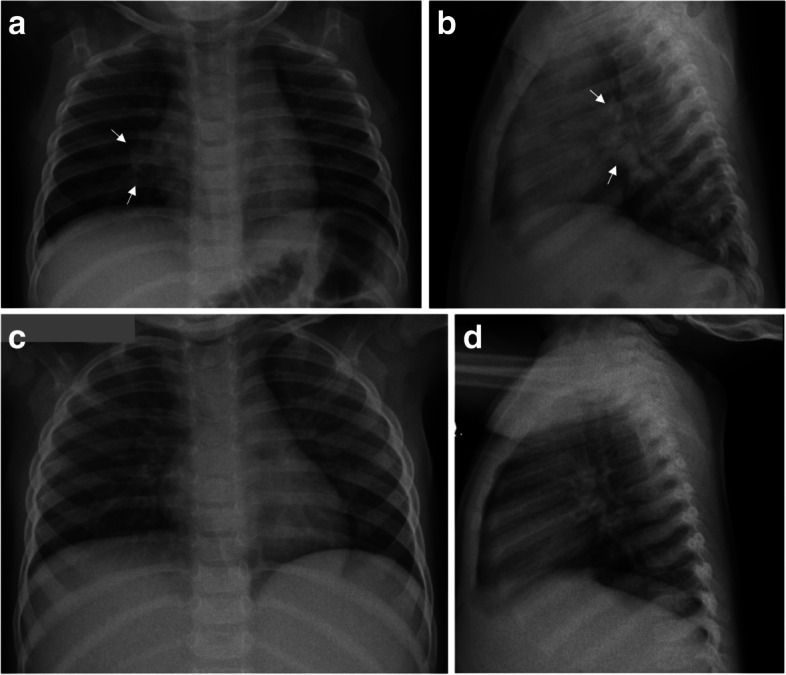
Normal and consistent with tuberculosis chest radiographs from children household contact of patients with tuberculosis. **a**,**b** Anteroposterior (**a**) and lateral (**b**) chest radiograph consistent with active tuberculosis in a 10-month-old boy. Round opacity parahiliary right suggestive of lymphadenopathy. **c**,**d** Normal anteroposterior (**c**) and lateral (**d**) chest radiograph in a 15-month-old girl

Every observer read twice between 94 and 110 chest X-rays. The 278 children participating in the study were predominantly male (*n* = 156, 56.1%), average age 31 months (SD 17), the responsible adult had incomplete secondary school (*n* = 75, 27%), socioeconomic stratum 2 (*n* = 133, 47.8%), BCG vaccination (*n* = 282, 96.6%), positive TST interpretation (cut-off point 5 mm) (*n* = 195, 70.7%) and were classified as infected at the time of recruitment (*n* = 203, 73%). The standardized reading of the chest radiograph was normal in 255 (91.7%) cases, consistent with TB in 10 (3.6%), and other alterations in 13 (4.7%). (Table 1, Fig. 1).

**Table 1:** Sociodemographic, exposure and clinical characteristics of children household contact of patients with tuberculosis

Characteristics	N	%
Sex		
Male	156	56.1
Female	122	43.9
Age in months Mean (SD)	31	17.0
Age (months)		
< 12	48	17.3
12–23	57	20.5
24–35	49	17.6
36–47	64	23.0
48–59	53	19.1
> 59	7	2.5
Municipality residence		
Medellín	264	95.0
Bello	9	3.2
Itagüí	5	1.8
Schooling of the person in charge		
Illiterate	12	4.3
Incomplete primary	14	5.0
Complete primary	25	9.0
Incomplete secondary	75	27.0
Completed secondary	65	23.4
Technique	68	24.5
Graduate or more	18	6.5
No data	1	0.4
Socioeconomic		
1	96	34.5
2	133	47.8
3	42	15.1
4	5	1.8
5	2	0.7
Overcrowding		
No	191	65.9
Yes	99	34.1
Displaced condition		
No	184	77.6
Yes	53	22.4
Health care affiliation		
Does not have	11	3.8
Linked	17	5.8
Subsidized	131	44.9
Contributory	129	44.2
Ethnicity		
Others	221	91.3
Indigenous	17	7.0
Afro-descendant	4	1.7
Relationship with the index case		
Son/daughter	77	27.7
Sibling	5	1.8
Cousin	5	1.8
Nephew/niece	86	30.9
Grandchild	64	23.0
Affinity relationship	7	2.5
Another degree of consanguinity	17	6.1
No relationship	17	6.1
Hours shared with the case index Mean (SD)	57	45.98
Smear of index case		
-	36	13.5
+	86	32.2
+ +	54	20.2
+ + +	91	34.1
BCG vaccine evidence		
Not vaccinated	2	0.7
Vaccinated	282	96.6
No card / No scar	7	2.4
No information	1	0.3
Persistent cough		
No	248	89.2
Yes	30	10.8
Weight loss		
No	273	98.2
Yes	5	1.8
Failure to thrive		
No	267	96.0
Yes	11	4.0
Unexplained fever		
No	272	97.8
Yes	6	2.2
Lethargy		
No	277	99.6
Yes	1	0.4
Lymphadenopathy		
No	277	99.6
Yes	1	0.4
Sweating		
No	269	96.8
Yes	9	3.2
Active TB consistent diagnosis		
No	233	83.8
Yes	45	16.2
QFT interpretation		
Negative	152	61.8
Positive	94	38.2
TST Interpretation (cut-off point 5 mm)		
Negative	81	29.3
Positive	195	70.7
Chest X-ray interpretation		
Normal	255	91.7
Consistent with TB	10	3.6
Other alterations	13	4.7
Initial infection status		
Infected	203	73.0
Immune window	69	24.8
Not infected	6	2.2
Prevalent active TB		
No	270	97.1
Yes	8	2.9

*SD* Standard Deviation, *BCG* Bacillus Clamette Guérin, *TB* Tuberculosis, *QTF* Quantiferon TB-Gold, *TST* Tuberculin Skin Test, *mm* millimeters

In the bivariate analysis, it was observed that overcrowding and ethnicity are related to a higher prevalence of radiological findings consistent with TB or other alterations (*P*-value likelihood ratio: 0.042 and 0.014 respectively). However, it was also observed that there is a higher prevalence of reading compatible with TB according to the following characteristics: indigenous ethnicity (PR = 8.78; 95%CI: 2.5—30.1), not having a health care affiliation (PR = 9.23; 95%CI: 2.0—41.1), crowded condition (PR = 3.91; 95%CI: 1.0—15.2), no evidence of BCG (PR = 6.37; 95%CI: 1.5—26.2), smoke exposure (PR = 3.55; 95% CI: 0.9—13.4), weight loss documented at clinical evaluation (PR = 7.25; 95%CI: 1.1–44.5), failure to thrive (PR = 6.37; 95%CI: 1.5—26.2) and sweating (PR = 7.11; 95%CI: 1.7—28, 8). (Table 2).

**Table 2:** Sociodemographic, clinical and exposure characteristics according to the interpretation of the chest X-ray in children household contact of patients with tuberculosis

Characteristics	N	Interpretation					
		**Consistent with TB**			**Other alterations**		
		**n**	**%**	**PR (95% CI)**	**N**	**%**	**PR (95% CI)**
Household contact sex							
Female	122	4	40	1	5	38.5	1
Male	156	6	60	1.18 (0.3—4.1)	8	61.5	1.25 (0.4—3.7)
Age (months)							
≥ 12	230	6	60	1	12	92.3	1
< 12	48	4	40	3.09 (0.9—10.5)	1	7.7	0.42 (0.1—3.1)
Ethnicity							
Others	217	5	62.5	1	11	100	1
Indigenous	14	3	37.5	8.78 (2.5—30.1)	0	0	/ *
Socioeconomic strata							
Not low	49	1	10	1	0	0	1
Low	229	9	90	2.04 (0.2—15.7)	13	100	5.98 (0.3—99.0)
Health care affiliation							
Contributory	126	3	30	1	6	46.2	1
Linked—Subsidized	139	4	40	1.21 (0.2—5.3)	7	53.8	1.06 (0.3—3.0)
Does not have—No data	13	3	30	9.23 (2.0—41.1)	0	0	/
Displacement condition							
No	177	5	62.5	1	8	72.7	1
Yes	50	3	37.5	2.15 (0.5—8.6)	3	27.3	1.37 (0.3—4.9)
Overcrowding							
No	185	3	33.3	1	11	84.6	1
Yes	91	6	66.7	3.91 (1.0—15.2)	2	15.4	0.38 (0.08—1.7) *
Proximity to the index case							
Does not live in the same house	78	2	20	1	3	23.1	1
Sleeps in the same bed or room or house	200	8	80	1.57 (0.3—7.2)	10	77	1.31 (0.3—4.6)
BCG evidence							
Vaccinated	268	8	80	1	13	100	1
Not vaccinated, no scar, no card, no information	10	2	20	6.37 (1.5—26.2)	0	0	/
Smoke exposure							
No	169	3	30	1	9	69.2	1
Yes	109	7	70	3.55 (0.9—13.4)	4	30.8	0.72 (0.2—2.3)
Persistent cough							
No	248	9	90	1	13	100	1
Yes	30	1	10	0.87 (0.1—6.6)	0	0	/
Weight loss							
No	273	9	90	1	12	92.3	1
Yes	5	1	10	7.25 (1.1—44.5)	1	7.7	5.50 (0.9—32.7)
Failure to thrive							
No	267	8	80	1	12	92.3	1
Yes	11	2	20	6.37 (1.5—26.2)	1	7.7	2.39 (0.3—16.5)
Sweating							
No	269	8	80	1	13	100	1
Yes	9	2	20	7.11 (1.7—28.8)	0	0	/
active TB consistent diagnosis							
No	233	7	70	1	12	92.3	1
Yes	45	3	30	2.15 (0.5—8.0)	1	7.7	0.44 (0.1–3.3)
TST Interpretation (cut-off point 5 mm)							
Negative	81	2	20	1	6	46.2	1
Positive	195	8	80	1.59 (0.3–7.3)	7	53.8	0.49 (0.1—1.4)

*BCG* Bacillus Calmette Guérin, *TB* Tuberculosis, *TST* Tuberculin Skin Test, *PR* Prevalence Ratio

^*^ *P*-value for likelyhood test < 0.05

When evaluating the difference in paired proportions (McNemar’s test), it was found that there was no difference between the proportions of the interpretation of the inter-observer or intra-observer reading (global inter-observer *P*-value = 0.077; inter-observer: pair 1–2 *P*-value = 0.129, pair 1–3 *P*-value = 0.172; intra-observer: reader 1 *P*-value = 0.082, 2 *P*-value = 0.543, for reader 3 was not possible to estimate the McNemar’s test).

Overall agreement (reader 1 vs reader 2 + reader 3) was 91.3% (kappa = 0.51; 95%CI: 0.4—0.7). The inter-observer agreement between reader 1 and reader 2 was 90% (kappa = 0.59; 95%CI: 0.4—0.7; *P* < 0.001); between reader 1 and reader 3 it was 93.2% (kappa = 0.59; 95%CI: 0.4—0.8; *P* < 0.001). The intra-observer agreement for reader 1 was 95.5% (kappa = 0.86; 95%CI: 0.8—1; *P* < 0.001); for reader 2 84% (kappa = 0.51; 95%CI: 0.3—0.7; *P* < 0.001); and for reader 3 was 94.7% (kappa = 0.68; 95%CI: 0.5—1; *P* < 0.001). The kappa value suggested a moderate agreement in the global, inter-observer and intra-observer agreement, except for the intra-observer agreement of reader 3, which was good and for intra-observer reader 1, it was very good (Table 3).

**Table 3:** Agreement of the interpretation of chest X-ray in children household contact of patients with tuberculosis

Characteristic	% Agreement	Kappa	95% CI	*P*-value
Global agreement	91.3	0.51	0.3- 0.6	< 0.001
Inter-observer agreement 1 and 2	90.0	0.59	0.4—0.7	< 0.001
Inter-observer agreement 1 and 3	93.2	0.59	0.3—0.8	< 0.001
Intra-observer agreement 1	95.5	0.86	0.7—0.9	< 0.001
Intra-observer agreement 2	84.0	0.51	0.3—0.7	< 0.001
Intra-observer agreement 3	94.7	0.68	0.4—0.9	< 0.001

*CI* Confidence interval

*P*-value for kappa coefficient

From the aspects evaluated in the chest radiograph, there was a high overall agreement, but there was some type of disagreement between reader pairs 1–2, and 1–3. The greatest disagreement was found in soft tissue density compatible with lymphadenopathy 4.6% and 4.2%, followed by airspace opacification 1.17% and 2.5%, respectively. Other discrepancies of reader 1 and 2 were in pleural effusion (0.58%) and reader 1 and 3 in cavities (0.8%). While the parameters with greater agreement were airway compression and/or trachea displacement (100%), disseminated and bilateral nodular = miliary or greater image (100%), pleural effusion (99.4%—100%), and cavities (100%). The kappa coefficient was only calculable in soft tissue density suggestive of lymphadenopathy (reader 1–2 kappa = 0.32; 95%CI:—0.0—0.6; *P*: < 0.001; reader 1–3 kappa =—0.01; 95% CI: -0.04—0.01; *P* = 0.851) and airspace opacification (reader 1–2 kappa = 0.49; 95%CI:—0.1—1.0; *P* < 0.001; reader 1–3 kappa = -0.71; 95% CI: 0.4—1.0; *P* < 0.001). (Table 4).

**Table 4:** Inter-observer agreement of the interpretation by item evaluated in the chest X-ray in children household contact of patients with tuberculosis

	**Aspect evaluated**		**Reader 2**			**Reader 3**		
			**No**	**Yes**	**% Disagreem**	**No**	**Yes**	**% Disagreem**
**Reader 1**	Airway compression and tracheal deviation	**No**	170	0	0	118	0	0
		**Yes**	0	0		0	0	
	Soft tissue density suggestive of adenopathy	**No**	161	8	4.6	113	4	4.2
		**Yes**	0	2		1	0	
	Opacification of airspace	**No**	167	0	1.17	111	3	2.5
		**Yes**	2	1		0	4	
	Nodular image = miliary or greater disseminated bilateral	**No**	171	0	0	118	0	0
		**Yes**	0	0		0	0	
	Pleural effusion	**No**	170	1	0.58	118	0	0
		**Yes**	0	0		0	0	
	Cavities	**No**	170	0	0	117	1	0.8
		**Yes**	0	0		0	0	

*Disagreem.* Disagreement

For intra-observer agreement by items, to reader 1 the kappa was calculable for soft tissue density suggestive of lymphadenopathy (kappa = 0.79; 95%CI: 0.4—1.0; *P* < 0.001), opacification of airspace (kappa = 1.0; 95%CI: 1.0–1.0; *P* < 0.001) and cavities (kappa = 1.00; 95%CI: 1.0—1.0; *P* < 0.001). For other items, there was 100% agreement, which does not allow the kappa to be calculated and the disagreement was low. For reader 2, kappa was calculable for soft tissue density suggestive of lymphadenopathy (kappa = 0.47; 95%CI: 0.1—0.7; *P* < 0.001) and airspace opacification (kappa = -0,01; 95%CI: -0.02—0.00; *P* = 0.9219), for other items there was also 100% agreement, which does not allow the kappa to be calculated. For reader 3, kappa was calculable for soft tissue density suggestive of lymphadenopathy (kappa = 0.38; 95% CI: -0.1- 0.9; *P* < 0.001) and airspace opacification (kappa = 0.86; 95%CI: 0.5—1.0; *P* < 0.001), there was 100% agreement in some items, it does not allow the kappa to be estimated (Table 5).

**Table 5:** Intra-observer agreement readers 1, 2 and 3 for chest X-ray of children household contact of patients with tuberculosis

	**Aspect evaluated**		**No**	**Yes**	**Agreem.%**	**Disagreem.%**
**Reader 1**	Airway compression and tracheal deviation	**No**	110	0	100	0
		**Yes**	0	0		
	Soft tissue density suggestive of adenopathy	**No**	107	0	99	0.9
		**Yes**	1	2		
	Opacification of airspace	**No**	105	0	100	0
		**Yes**	0	5		
	Nodular image = miliary or greater disseminated bilateral	**No**	110	0	100	0
		**Yes**	0	0		
	Pleural effusion	**No**	110	0	100	0
		**Yes**	0	0		
	Cavities	**No**	109	0	100	0
		**Yes**	0	1		
**Reader 2**	Airway compression and tracheal deviation	**No**	106	0	100	0
		**Yes**	0	0		
	Soft tissue density suggestive of adenopathy	**No**	91	5	91.4	8.5
		**Yes**	4	5		
	Opacification of airspace	**No**	104	1	98.1	1.88
		**Yes**	1	0		
	Nodular image = miliary or greater disseminated bilateral	**No**	107	0	100	0
		**Yes**	0	0		
	Pleural effusion	**No**	106	0	99	0.9
		**Yes**	1	0		
	Cavities	**No**	105	0	99	0.9
		**Yes**	1	0		
**Reader 3**	Airway compression and tracheal deviation	**No**	94	0	100	0
		**Yes**	0	0		
	Soft tissue density suggestive of adenopathy	**No**	90	0	96.8	3.2
		**Yes**	3	1		
	Opacification of airspace	**No**	84	3	96.8	3.2
		**Yes**	0	7		
	Nodular image = miliary or greater disseminated bilateral	**No**	94	0	100	0
		**Yes**	0	0		
	Pleural effusion	**No**	94	0	100	0
		**Yes**	0	0		
	Cavities	**No**	93	0	98.9	1.1
		**Yes**	1	0		

*Agreem.* Agreement, *Disagreem.* Disagreement

^*^Kappa index cannot be calculated because all readings were normal, consistent

Image quality of 8 AP chest X-rays and 13 lateral were rated as poor but readable by at least one of the readers, and 1 lateral was rated as not acceptable, not readable by one of the readers, remaining chest X-rays were rated as acceptable by all readers.

## Discussion

TB is an infectious disease that occurs in all ages, with high incidence and prevalence worldwide. It can directly affect children under the age of five, contacts of patients with pulmonary TB, causing the risk of becoming infected and developing the disease throughout life. For this reason, this age group requires special attention and vigilance for an early diagnosis to provide timely preventive treatment and prevents the progression to disease immediately or in subsequent years.[3, 17, 18, 23, 24]. In developing countries like Colombia, access barriers to health care services are high, and basic diagnostic tools such as chest radiography are needed to make an accurate diagnosis. It has a direct impact on the diagnostic process of this disease, by contributing to the classification of latent or active TB after the infection has been confirmed [2, 25, 26].

The population group studied was characterized by being mostly from Medellín, having a predominance of males, an average age of 31 months, belonging to low socioeconomic strata and a significant percentage living in crowded and displaced conditions. The majority were infected at the beginning with positive TST (cut-off point 5 mm). Furthermore, the global interpretation of the initial chest X-ray concluded that the majority were normal but evidenced suspicion of active TB in a significant percentage (6.7%), demonstrating the direct link of the disease with difficult economic situations. Guarda and Kreft claim that to achieve the diagnosis in children, a chest X-ray is required, which is a fundamental tool for the diagnostic support of these patients. What’s more, they clarify that a normal chest X-ray does not rule out the presence of lung disease; therefore, the reading of the radiograph must be carried out in a very detailed way, thus considering any finding as suspicious of the disease [23, 27].

In a study published in Indonesia in 2015, the evaluation of chest radiograph was proposed in the context of detection of childhood TB contacts in the community, describing the quality and inter-observer findings. Concluding that chest radiograph of contact children with TB investigated in the community, were characterized by low quality (kappa: 0.16—0.35) and low agreement in reading normal radiographs (agreement between observers, kappa: 0.25–0.46) and low agreement in the most common pathological finding (inter-observer agreement, kappa: 0.03–0.25). Because image quality is generally poor in sites where resources are limited, identification and interpretation of abnormalities on chest radiograph are variable and often inconsistent [16]. It is important to highlight that in this study a standardized protocol was not carried out; the radiographs were read by two pediatricians and two radiologists and were not read by a third person in case of being discordant to verify their result. When comparing these results with the findings of the present study, it was found that when a standardized reading protocol is used, the strength of the agreement and the reliability of the X-ray reading could be increased, given a moderate strength of agreement according to the kappa coefficient, good and very good. This indicates that strategies are required to increase the agreement between the readings.

Standardized reading of the chest radiograph studies have been published, with the idea of unifying concepts. Graham et al. published in 2015 a standardized protocol for reading the chest radiographs in children with TB, developed by an international panel of TB experts; the authors concluded that reading this diagnostic tool continues to be a challenge, mainly in immunosuppressed children [10, 11].

In 2010, a study carried out in South Africa was published where authors implemented the use of a standardized reading template for chest radiographs in pediatric TB patients, it was found that the use of the template strengthened and guided the diagnosis of the disease [21].

Ominde et al. in 2018 reported the agreement of the reading of chest radiographs performed according to the WHO standard indications, in children who received pneumococcal conjugate vaccine to estimate the efficacy of vaccination. A total of 2716 chest radiographs were interpreted initially by two physicians (primary readers), with a second reading of discordant chest radiographs plus 13% of concordant ones, which were read again by a panel of three expert radiologists. With percentages of agreement and kappa for radiologically confirmed pneumonia (RCP) of 89% and 0.68, which ranged between 84%—97% and 0.19—0.68, respectively, for all pathological findings. The sensitivities of the primary readers to detect RCP were 69% and 73%, and the specificities were 96% and 95%. Concluding that the intra-observer and inter-observer agreement in the interpretation of RCP varies from fair to good, with moderate sensitivity and high specificity. Thus, demonstrating that the use of a reading protocol for chest radiograph was an adequate measure to evaluate the relative effectiveness of the vaccine. However, the reading of the radiograph remains a challenge that requires more strategies that improve the agreement of the readings [28].

In 2013, Xavier-Souza et al. evaluated inter-observer agreement in 803 chest radiographs of children aged 2 to 59 months included in a trial for amoxicillin use. One of the inclusion criteria was the presence of pulmonary infiltrate on the chest radiograph. Initially the chest radiographs were read by a pediatrician and later by two pediatric radiologists independently and blinded; the interpretation was standardized with the WHO guidelines. The result of the kappa coefficient for the presence of pneumonia was 0.725 (95%CI: 0.675–0.775), with an overall agreement of 78.7% (normal radiography [*n* = 385, 60.9%], pneumonia [*n* = 222, 35.1%], another radiological diagnosis [*n* = 22, 3.5%] and inappropriate for reading [*n* = 3, 0.5%]). Agreement for consolidation was 86.7% (kappa = 0.683, 95%CI: 0.631–0.741). Concluding that the agreement was good between two pediatric radiologists when comparing the diagnosis of pneumonia among children with acute non-serious lower respiratory tract infection. Even though this study did not have a standardized protocol and with a third reader for the cases with disagreement, it did have a standardized interpretation according to the WHO guidelines; the agreement was good and comparable with the findings of the present study. This suggests that having X-ray reading protocols may be associated with a kappa coefficient that indicates better agreement [29].

In addition to this, large studies have shown errors in the non-standardized reading of different radiographs (chest, spine, hip, knee, ankle, and foot), in 1996 Brunswick et al. published a study on the interpretation of radiographs from a community hospital, which were initially read by emergency physicians and interpreted a second time by hospital radiologists. They reviewed 15,585 radiographs, of which 99% were read correctly at the initial reading. The global disagreement of the readings obtained was 1% (*n* = 121), of which 5.7% (*n* = 7) were overinterpreted, 47.1% (*n* = 57) under interpreted with no changes in patient treatment and 47.1% (*n* = 57) had a wrong reading. Which shows that there can be a margin of error in the reading of radiographs when standardized readings are not used [30].

Within the sociodemographic, exposure and clinical characteristics of included children in this study, the majority had a status of infected at the beginning, with a normal chest X-ray; reflecting the high possibility of becoming infected when having contact with an adult with TB and the urgent need to receive preventive treatment in these cases, to prevent active TB after infection. The essential function of chest radiograph in these patients is the cornerstone of this study. The results showed that the intra-observer agreement may vary and not be 100% reliable since it is dependent on the reader; in addition to this, the image observed in X-rays has two dimensions, which increases the difficulty of diagnosis, comparing to other type of image tools as the computed tomography scan. These concepts explain the need for standardized protocols for reading chest radiographs in these patients to unify reading concepts and avoid diagnostic errors and delays in the beginning of the treatment. The inter-observer agreement evaluated between the different readers shows that it can increase the agreement and lead to more reliable readings to suspect or not the diagnosis of active TB in these children. For this reason, this study invites new research to contribute to this issue, since they are fundamental in reducing the incidence of active TB in childhood.

In the present study, it was observed that the global, intra-observer and inter-observer agreement were high and greater compared to those found in the studies described in the literature; however, there was some type of disagreement although it was low. In addition to this, the estimated kappa coefficients for the agreement were equal to or higher to those found in other studies. For this reason, it is important to have adequate training, within the framework of standardized protocols for reading chest radiographs, which can contribute favorably to the timely diagnosis of latent TB in children under the age of five, who had contact with adult patients with pulmonary TB.

The data gathered was low for the specific characteristics in the radiograph reading, which limits the possibility of building a multivariate model that fits adequately. Although chest radiographs play a very important role that directly contributes to the diagnosis of TB in exposed population, the reading is operator dependent, the image has two dimensions, and the quality of the technique is not always the best, which can produce a subjective result that leads to delays or misdiagnosis. Further studies that contribute to promoting the standardized reading of chest radiograph in children with contact of adults with TB are needed to increase the possibility of unifying concepts in reading.

## Conclusions

This study found that inter-observer and intra-observer agreement for chest radiograph reading ranged from moderate to very good, showing that having standardized protocols for reading the chest radiograph in the pediatric population under the age of five years exposed to TB, can increase the reading agreement leading to a more reliable results for diagnosing TB in childhood. The pediatric population exposed to TB presents difficult conditions due to its low socioeconomic and educational level, which increases the barriers to access to health care services and preventive treatments, making the chest radiograph a very useful and valid tool to support TB assessment in this population, giving value to standardized protocols for its reading.

## Data Availability

The datasets used and/or analyzed during the current study are available from the corresponding author on reasonable request.

## Abbreviations

AP: Anteroposterior
BCG: Bacillus Calmette Guerin
CI: Confidence interval
HIV: Human immunodeficiency virus
PR: Prevalence ratio
QTF: Quantiferon TB-Gold
RCP: Radiologically confirmed pneumonia
SIVIGILA: Epidemiological surveillance system in Colombia
TB: Tuberculosis
TST: Tuberculin skin test
WHO: World Health Organization
